# Acute Cerebral Palsies in Children

**Published:** 1897-02

**Authors:** S. G. Courtney Pinckney

**Affiliations:** Atlanta, Ga., Lecturer on Diseases of the Nervous System, Southern Medical College


### ACUTE CEREBRAL PALSIES IN CHILDREN.

By S. G. COURTNEY PINCKNEY, Atlanta, Ga.,

Lecturer on Diseases of the Nervous System, Southern Medical College.

Children are liable to acute paralysis. As in adults, the lesion may be in the nerves, cord, or brain; the nerves rarely suffer, the cord frequently. It is, however, only of palsies of cerebral origin that I wish to speak.

The word hemiplegia (or paralysis of one-half of the body) has lost its strict meaning and is used to designate the paralysis of cerebral origin, no matter what the location is in the extremities.

The lesion is in the motor area, either in the cortex, brain substance or at the base; identical lesions can and do occur elsewhere in the brain, but by compensating development the other hemisphere takes up the function of the destroyed part so that permanent symptoms, unless the motor or optic tracts are involved, do not remain. On autopsy brains have been found containing large cavities with no suspicion of brain disease during life.

Though acute cerebral palsies are not at all uncommon, opinions as to their causation are contradictory. In spite of the gravity and permanency of the damage done, the lesion is not often fatal. With the exception of thrombotic softening the tendency is towards partial recovery, with not very much danger to life; for this reason the pathological condition is rarely seen till many years after its onset, when the appearance of the lesion has materially changed.

I here only consider acute palsies—those with an onset of a few hours or a day. More chronic processes, like tumors, or even abscesses, are not considered.

It is to the vascular apparatus we look as a cause for the greater number of these cases. Some, however, do not fit in this category, and it is of these cases I particularly wish to speak later.

About 50 per cent. of these cases occur in the first year of life and 20 per cent. in each of the two succeeding years. The parts affected are the leg and arm of one side—the leg, arm and face, or the arm alone—rarely both legs alone.

These palsies have much in common—an acute onset with cerebral symptoms, followed by a spastic palsy with increased reflexes, stiffening and contractions. There are no trophic or sensory symptoms. The history given us for diagnosis is meagre enough; the mother tells us that the child vomited or had a convulsion, became unconscious, and in an hour or day was paralyzed.

On examining the child we find one of the conditions before mentioned. The vascular lesions are the same as those occurring in adults, with symptoms modified by the age of the patient; it is convenient to classify them in the same way—hemorrhage, embolism and thrombosis.

*Embolism* of the middle cerebral artery is the most common of these lesions, either the main trunk or one of its branches being plugged. The source of the embolus is the heart or the arch of the aorta; endocarditis can generally be found.

We have in these cases a sudden onset of coma, its duration depending on the size of the vessel plugged. When consciousness returns it is seen that the child is paralyzed, the paralysis corresponding with the extent of cortex deprived of blood; later come athetotic movements and local convulsions. Fever, vomiting or prodromal symptoms are *not* to be expected.

*Hemorrhage* is the rarest of all lesions; it is impossible to diagnose a hemorrhage of the cortex from embolism. On the other hand, lesions of the internal capsule are generally hemorrhagic. Practically, the history is the same as embolism. We are not warranted in diagnosing hemorrhage if a heart lesion exists.

*Thrombosis* of an artery is practically always secondary to embolism; the history is one of embolism.

*Thrombosis* of a sinus is a most serious disease; the superior longitudinal sinus is nearly always the one affected; though I know of no statistics published, the mortality must be at least 80 per cent. It is generally the result of exhausting diarrheas, wasting diseases as tuberculosis, or the infectious fevers of childhood. As these are often fatal themselves, it is hard to get the mortality; it is, however, great.

Different as these pathological conditions are, if seen after the onset, the appearance is nearly identical. On examination of the child, we find a paralysis of from one limb to an entire side, spastic stiffening, athetotic movements and increased reflexes. Later we have contractures and arrested development of the limb, and if the disease is cortical, recurring attacks of Jacksonian epilepsy. Careful questioning is necessary to arrive at a diagnosis, and from the history thus given we work out the lesion.

*Embolism.*—The source of the embolus is generally the heart, acute or more rarely chronic endocarditis; occasionally it comes from the aorta or lungs. In typical cases we would expect—

An acute onset, acute attack of unconsciousness without warning, spasmodic twitching in affected limbs, coma never very prolonged, often only a few minutes.

Temperature normal or depressed one-half degree.

Aphasia, if right side is paralyzed.

Cranial nerves unaffected.

Reflexes at first inhibited; later much increased.

Later we have attacks of Jacksonian epilepsy in affected limbs, athetotic movements, contractures and retarded development.

As a rule, only one limb is permanently paralyzed.

The picture is one of an acute though fairly mild onset, followed by permanent though limited symptoms of irritative paralysis later.

*Hemorrhage.*—We have the same acute onset, but the coma is much deeper and more prolonged. A very small hemorrhage will cause more severe symptoms at the onset than the largest embolus possible.

As nearly all hemorrhages occur at the base from rupture of the lenticulo-striate artery, we do not have the symptoms of cortical irritation. Though the limb is paralyzed and somewhat stiff there is neither convulsions, athetosis or spasm of the paralyzed part. On the other hand, as the damage is in the internal capsule, the paralysis is much more extensive, generally involving a whole side.

The temperature falls in an hour from one to four degrees.

No aphasia.

After the coma disappears the condition is almost unchanged.

Later we have a persistent paralysis and stiffness.

The picture is one of an acute and severe onset followed by extensive and permanent results of a passive, instead of irritative nature.

In sinus thrombosis the picture is entirely different.

*Thrombosis* may be primary or secondary. Secondary thrombosis occurs from extension of suppurative processes near the sinus, generally in the lateral or cavernous sinus, as a result of ear disease. It gives no marked motor symptoms, so does not concern us here.

Primary thrombosis is the result of certain blood states, resulting in increased coagulability of the blood or diminished blood pressure, such as the infectious fevers, tubercular disease, or exhausting intestinal disease. Hemorrhage has caused this condition by reducing the volume of blood; the rigid sinus walls are unable to contract, so the blood current is very much slower.

In all cases of which I have notes the superior longitudinal sinus was affected.

We first have a history of the exhausting disease, prolonged diarrhea or bad health; then some day general convulsions, slight or severe, occur, followed by apathy, somnolence or coma. Vomiting is an early symptom, and if the child is old enough to talk, headache and mental dullness may be noticed a day in advance.

In from a few hours to a day there are developed internal strabismus, stiffness of the neck muscles, rigidity of the limbs, either both arms or both legs; epistaxis, prominence of fontanelle and edema of the scalp.

There are recurring convulsions through the whole acute process, often every half hour; these are general. Unilateral convulsions, when they exist, are due to the extension of the clot into the surface veins of the cortex.

With even such severe symptoms there is little or no rise of temperature; it is either normal or that already existing.

The prognosis is bad; fully 80 per cent. die. If recovery takes place there is the same spastic paralysis that occurs with embolism and the same irritative cortical symptoms, Jacksonian spasm and contractures, mental dullness and imperfections.

The whole picture is one of an acute cerebral process, very suggestive of meningitis, supervening on some exhaustive disease, having an onset of from an hour to a day and characterized by repeated convulsions.

All these cases have been the result of vascular disease. They are non-febrile in themselves, and inflammatory results are slight and always secondary to them. There still exists a class of cases decidedly different in onset, though resulting in the same variety of paralysis, the etiology of which is yet involved in doubt.

A healthy child is suddenly taken ill with severe meningeal symptoms; there is a rapid rise of temperature to 103 or more degrees, vomiting, general convulsions, restlessness or stupor. In twenty-four hours these symptoms diminish, and in forty-eight have, as a rule, disappeared. In a day or so it is noticed that the child is partially paralyzed on one side, face, arm and leg. This increases for a week, and then slowly improves a little, and in a year some use of the limbs is regained.

After recovery we have all the signs of a cortical lesion. Spastic paralysis, athetotic movements and attacks of Jacksonian spasm.

There is no involvement of the cranial nerves, but if the left side of the brain is affected aphasia is present.

These cases differ from the other types mentioned decidedly.

The picture is one of acute febrile disease running its course, followed after a short interval by paralysis.

These symptoms have long been attributed in a vague way to an acute encephalitis, a localized primary inflammation of the brain corresponding to a myelitis in the cord.

Strümpell has very decided ideas that are worthy of more attention than they have yet received. He believes these cases to be due to an inflammation of the motor cortex cells, a polioencephalitis, a condition with the same etiology and pathology as poliomyelitis.

This theory, though accepted in Germany, is not credited elsewhere.

Gowers sums up the following objections to it:

1. A primary inflammation limited to certain cells in a certain part of the cortex is theoretical and scarcely possible.

2. If it did occur we would have some pathological examples of the disease in the acute stage, as diseases of the brain are more fatal than those of the cord.

3. That the liability of the brain to disease is very much less than the cord, and we would expect infection to attack the cord by preference.

These objections seem to me readily answered.

That the condition is theoretical is admitted, since no specimens have been secured during the acute stage. Autopsy in later life has, however, shown the identical changes found in old poliomyelitis cases, disappearance of cellular elements, increase of connective tissue, induration and shrinking of the affected part of the cortex; in each variety of tissue the same changes.

To the objection that as diseases of the brain are more fatal than those of the cord, so, therefore, we should have some specimens showing acute changes; the answer would be that the far greater frequency of cord disease more than evens this up, and that as an actual fact this disease, whether in cord or brain, is very rarely fatal.

As to the lesser liability of the brain, this disease, when it occurs in the cord, does not affect the whole tract, but picks out situations often widely apart. If it could affect in one person the lumbar segment and in another the cervical, it might readily affect the cortex in a third case.

The treatment must vary in each condition.

In hemorrhage we do absolutely nothing. Any interference is dangerous; if the heart action becomes weak, strophanthus in small doses to strengthen it. Never use digitalis or strychnine—absolute quiet in a darkened room, on a milk diet.

In embolism the symptoms are due to the cortical anemia, resulting from the plugging of the artery. If we can push the plug farther along the artery we diminish the number of branches cut off. To do this we give nitro-glycerine and caffeine, so dilating the artery and stimulating the heart. Nitro-glycerine is not in itself a heart stimulant. Quiet, with a simple and nourishing diet.

*In thrombosis* stimulation is of the greatest importance. In secondary thrombosis opening and packing the diseased sinus has given the best results, but I have never seen a case of primary thrombosis in which surgical procedure was practicable. The original disease has generally caused such great depression that surgical interference was unwarranted. All the cases I have seen so far have been fatal.

*In polioencephalitis* we evidently have an acute febrile disease with an inflammation of the cortex as the characteristic lesion. The management is that of other febrile conditions. Small doses of aconite, belladonna and brandy, rest and tepid baths are best.

The resulting contractures, spasms and paralysis are best treated by galvanism. Much is said against electrical treatment, principally by physicians who possess a small farradic battery, and who are unacquainted with the difference between a primary and secondary current. The effects of proper electrical treatment are often striking.

Massage and passive motion are subsidiary measures.

For the Jacksonian spasms bromides are best, but hot baths are also useful.

Slight as the mortality is, it would be still smaller were it not for the fact that all these cases are generally mistaken for meningitis, and depletive measures, assisted by cold to the head, is the staple treatment. The weaker children promptly succumb, but the stronger manage to pull through.

Counter irritation in any form is useless.

*302 Peachtree Street.*

